# Comparison between small radiation therapy electron beams collimated by Cerrobend and tubular applicators

**DOI:** 10.1120/jacmp.v16i1.5186

**Published:** 2015-01-08

**Authors:** Cristina Di Venanzio, Marco Marinelli, Alessia Tonnetti, Gianluca Verona‐Rinati, Paolo Bagalà, Maria Daniela Falco, Antonio Stefano Guerra, Maria Pimpinella

**Affiliations:** ^1^ INFN–Dipartimento di Ingegneria Industriale Università di Roma Tor Vergata Rome Italy; ^2^ Department of Diagnostic Imaging Molecular Imaging, Interventional Radiology and Radiotherapy, Tor Vergata University General Hospital Rome Italy; ^3^ Istituto Nazionale di Metrologia delle Radiazioni Ionizzanti ENEA‐INMRI C R Casaccia Rome Italy

**Keywords:** Cerrobend, tubular applicators, diamond, radiation therapy, small electron beams

## Abstract

The purpose of this study was to compare the dosimetric properties of small field electron beams shaped by circular Cerrobend blocks and stainless steel tubular applicators. Percentage depth dose curves, beam profiles, and output factors of small‐size circular fields from 2 to 5 cm diameter, obtained either by tubular applicators and Cerrobend blocks, were measured for 6, 10, and 15 MeV electron beam energies. All measurements were performed using a PTW microDiamond 60019 premarket prototype. An overall similar behavior between the two collimating systems can be observed in terms of PDD and beam profiles. However, Cerrobend collimators produce a higher bremsstrahlung background under irradiation with high‐energy electrons. In such irradiation condition, larger output factors are observed for tubular applicators. Similar dosimetric properties are observed using circular Cerrobend blocks and stainless steel tubular applicators at lower beam energies. However, Cerrobend collimators allow the delivery of specific beam shapes, conformed to the target area. On the other hand, in high‐energy irradiation conditions, tubular applicators produce a lower bremsstrahlung contribution, leading to lower doses outside the target volume. In addition, the higher output factors observed at high energies for tubular applicators lead to reduced treatment times.

PACS number: 87.53.Bn, 87.55.Qr, 87.56.Fc

## I. INTRODUCTION

Due to their sharp dose falloff and well‐defined ranges, megavoltage electron beams are used in the treatment of superficial neoplasms. According to the shape of the tumor, electron beam collimation is commonly achieved using standard applicators attached to the accelerator head. In the case of small targets, special collimators may be used in order to properly conform the beam and to protect healthy tissues or critical organs surrounding the tumor.^(1)^ Such collimator systems can be preconstructed and ready‐to‐use, as in the case of commercial cones or custom cut‐out shapes, placed at the end of a standard electron applicator. The former ones consist of a main tubular electron applicator fastened directly to the linac head and a set of add‐on field defining end tubes with a diameter ranging from 2 to 5 cm. The custom cut‐outs are usually made by Cerrobend (Cerro Metal Products Company, Bellefonte, PA), a low melting point alloy that can be easily molded into various sizes and forms and placed at the end of standard electron beam applicators. The thickness of the Cerrobend block is usually chosen to reduce the dose under the block by 95%–98% of the open beam dose.

Such applicators are expected to affect output factors (OFs), percentage depth dose curves (PDDs), and the shape of beam profiles. In particular, the lack of lateral scatter equilibrium occurring in small beam sizes^(2)^ results in a shift towards the phantom surface of the depth of maximum dose in the PDD curves.^(3)^ Additionally, a contribution from scattered and bremsstrahlung radiation generated by the collimator systems is expected.^4^, ^5^ Depending on the shape and material of the used collimator, such contribution can affect PDDs and profiles, as well as the OF values. A large dependence of OF values on the field shape and size has been reported in literature.^6^, ^7^

The aim of this work is to compare the dosimetric properties of small circular fields shaped by home‐made Cerrobend blocks and commercial tubular applicators in high‐energy electron beams.

To this purpose, a reliable tissue‐equivalent detector is needed, characterized by a high spatial resolution capability.^(8)^ A synthetic single crystal diamond diode (SCDD) was chosen for the present study, which was already demonstrated to be a suitable detector for small field sizes electron dosimetry.^(9)^

## II. MATERIALS AND METHODS

Dosimetric measurements of small‐size circular fields from 2 to 5 cm in diameter, obtained either using tubular applicators or Cerrobend blocks, were performed for 6, 10, and 15 MeV electron beams generated by an Elekta Precise linear accelerator (Elekta Crawley, UK) at Tor Vergata University General Hospital in Rome.

The tubular applicators consist of a main applicator fastened to the head of the accelerator and a set of add‐on interchangeable field‐defining end tubes producing field diameters of 2, 3, 4, and 5 cm at 100 cm distance from the source (Elekta Crawley, UK). All of them are made of stainless steel.

Small circular fields 2, 3, 4, and 5 cm in diameter were also produced using home‐made cut‐outs. The blocks were made of Cerrobend — an alloy consisting of bismuth (50%), lead (26.7%), tin (13.3%), and cadmium (10%), with a melting temperature of approximately 70°C and a density of about 9.4 g/cm^3^ at 20°C. The usual procedure was carried out for their production.^(10)^ The mold of the Cerrobend block was modeled in 1 cm thick Styrofoam. Finally, an electron beam shaping system (Aktina Medical, Congers, NY) was used to fabricate the custom low‐melting alloy blocks. Such blocks were subsequently attached to the end of $6\times 6\;{\text{cm}}^{2}$ electron standard applicator, at a distance of about 5 cm from the phantom surface. It's worth pointing out that the Cerrobend block tends to sink in the middle when cooling during the fabrication process, thus producing a thickness gradient between the edges and the center. The block thickness was verified by a digital caliper and found to be 1.1±0.1 cm. In addition, the blocks exhibit a not‐perfectly‐circular aperture. The actual diameter of the apertures was measured and the obtained average values were 1.9 cm, 2.8 cm, 4.0 cm, and 4.5 cm, respectively.

All dosimetric data were collected by using a PTW microDiamond 60019 premarket prototype (PTW, Freiburg, Germany), developed at the Industrial Engineering Department of the “Tor Vergata “ University of Rome in conjunction with PTW Freiburg.^9^, ^11^, ^12^, ^13^, ^14^

A PTW MP3 motorized water phantom was used for the measurements, at a SSD of 100 cm. Beam profiles and depth dose curves were acquired and analyzed through a PTW TANDEM electrometer and the PTW Mephysto ${\text{MC}}^{2}$ software, respectively. For the OF measurements, a PTW UNIDOS E Universal Dosimeter was used.

The detector was positioned with its main axis parallel to the electron beam direction. For each field size, both in‐plane and cross‐plane beam profiles were measured, and the detector was positioned at the center of the profiles to perform OF and PDD measurements.

PDD curves were measured along the central axis of each field size for all the beam energies. The depth in water corresponding to the maximum absorbed dose and the 50% of it, $R_{100}$ and $R_{50}$ respectively, were evaluated. The dose contribution due to bremsstrahlung was evaluated in terms of the $D_{x}$ parameter, defined as the PDD value at the depth of the maximum electron range $R_{\text{max}}$.^(15)^

Beam profiles, both in‐plane and cross‐plane, were measured for each circular field for 6, 10, and 15 MeV electron beams, at $R_{100}$ as determined by the PDD measurements. The profiles measured by using the two different collimator systems were compared in terms of 80%‐20% penumbra.

Finally, OF measurements were carried out at $R_{100}$ for all beam energies and circular fields, using the $10\times 10\;{\text{cm}}^{2}$ field as a reference.

## III. RESULTS & DISCUSSION

In this work, the differences in PDDs, OFs, and dose profiles were evaluated for small circular fields shaped both by tubular applicators and Cerrobend shielding blocks. All measurements were performed by using the premarket prototype of the PTW microDiamond dosimeter, as employed by Bagalà et al.^(9)^

**Figure 1:**
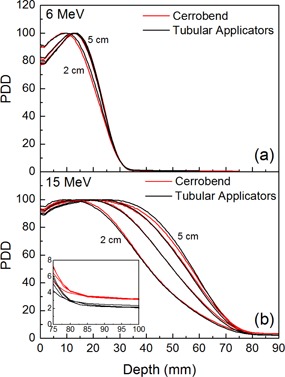
PDD curves measured for all the investigated field size diameters in 6 and 15 MeV electron beams shaped by Cerrobend blocks and long tubular applicators. The inset in (b) shows a magnification of the bremsstrahlung tails.

The PDD curves measured for both the tubular applicators and Cerrobend apertures are reported in Fig. 1 for all the investigated field sizes, under irradiation with 6 MeV (Fig. 1(a)) and 15 MeV (Fig. 1(b)) electron beams. The $R_{50}$ and $R_{100}$ values, together with the relative dose due to the bremsstrahlung tail, $D_{x}$, are reported in Table 1 for all the investigated beam energies and collimators. An overall similar behavior between the two collimating systems can be observed even though the effective aperture size is not directly comparable in all cases (see Table 1). A shift towards the phantom surface of $R_{100}$ and $R_{50}$ can be noticed by decreasing the field size, which is definitively more evident for the higher energies as already reported in literature.^(16)^ This can be explained in terms of lack of lateral scatter equilibrium occurring in small fields, as discussed in Bagalà et al.^(9)^ In the case of 15 MeV, higher $D_{x}$ values are measured for Cerrobend shielding blocks, indicating the presence of a higher bremsstrahlung component when such collimators are used. Such a component is found to be negligible at 6 MeV, being continuously increasing with beam energy as expected.^(4)^

**Figure 2:**
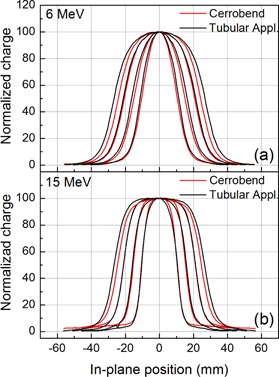
In‐plane normalized beam profiles measured in 6 (a) and 15 (b) MeV electron beams with field size diameter from 2 cm to 5 cm obtained using both Cerrobend blocks and long tubular applicators.

Figure 2 shows in‐plane normalized beam profiles in 6 MeV and 15 MeV electron beams. The 80%–20% penumbra values, as well as the measured field sizes (FWHM), are also reported in Table 1 for all the beam energies. Comparable results in terms of 80%—20% penumbra values are obtained both for the Cerrobend collimators and tubular applicators. In addition, for both the collimating systems, the measured FWHM is compatible with the actual collimator diameter. However, it can be noticed that under 15 MeV beam, the Cerrobend collimators produce a higher dose background outside the irradiation field. Such a background is not observed at lower energies. This is consistent with the above discussed higher bremsstrahlung background observed in the PDD measurements.

**Figure 3:**
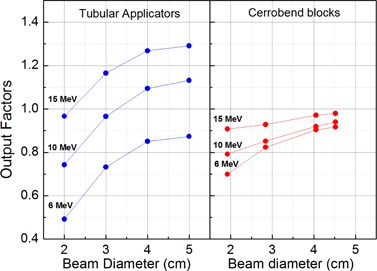
Relatives OFs versus field size diameter using both Cerrobend blocks and tubular applicators in 6, 10, and 15 MeV electron beams.

The OF values for all field sizes and energies are shown in Figs. 3(a) and 3(b) for Cerrobend shielding blocks and tubular applicators, respectively. In the case of tubular applicators, OFs larger than unity are observed at 10 MeV and 15 MeV, with an increase for the 5 cm diameter field size at 15 MeV up to 30% in comparison with the output of the $10\times 10\;{\text{cm}}^{2}$ field size. A decrease of the OF value is observed for each field size by decreasing the beam energy. A decrease of OF can also be noticed when reducing the field size below 5 cm diameter for a fixed energy. This behavior for the tubular applicators was explained by Bagalà et al.^(9)^ in terms of scattering of electrons both by air and by the collimator walls into the useful beam. In the case of Cerrobend shielding blocks, the typical OFs behavior reported in literature is observed instead.^2^, ^17^ In particular, the OF values are always less than unity, and the above‐described strong dependency on both the beam energy and field size is definitely reduced. Indeed, the extension of the applicator wall surface exposed to the electron beam is much less in the case of Cerrobend collimators. This implies that the effect of the electron scattering by the collimator walls is drastically reduced, leading to a less‐pronounced variation of the central axis electron fluence as a function of the field size. As can be seen in Fig. 3, the variation in OFs from the largest to the smallest field diameter is between 8% and 20% for the Cerrobend blocks and about 35% for the tubular applicators, irrespective of the beam energy. Moreover, Fig. 3 shows that the output factors are, in general, larger for the tubular applicators in comparison with the Cerrobend collimators, except for the 6 MeV electron beam and the 2 cm beam diameter at 10 MeV. In this regard, it should be considered that at low energy, electrons impinging on the walls of the long stainless steel tubular collimator are to a large extent absorbed and not scattered in the useful beam, as occurs at higher energies. This reduces the electron fluence on the beam central axis and then the absolute collimator output. This reduction effect is lower in the case of Cerrobend collimators, since the Cerrobend walls have a smaller length (about 1 cm against about 40 cm), so that the absorption of electrons in the collimator walls takes place to a lesser extent and laterally air scattered electrons contribute to the central axis fluence also at low energy.^15^, ^18^

**Table 1:** Parameters extracted from the PDD curves ($R_{100},R_{50}$, and $D_{x}$) and beam profiles (penumbra and FWHM) measured for electron beams shaped by Cerrobend blocks and tubular applicators

*Collimator Type*	*Cerrobend*	*Cone*	*Cerrobend*	*Cone*	*Cerrobend*	*Cone*	*Cerrobend*	*Cone*
*Aperture Diameter (cm)*	*1.92*	*2*	*2.84*	*3*	*4.05*	*4*	*4.52*	*5*
*6 MeV*								
$R_{100}\;(\text{mm})$	9.0	9.1	12.5	13.0	13.0	13.0	13.0	14.3
$R_{50}\;(\text{mm})$	21.9	22.8	23.4	23.6	23.7	23.8	23.6	24.1
$D_{x}\;(\%)$	0.9	1.1	0.9	0.8	0.8	0.7	0.9	0.9
Penumbra (mm)	8.5	7.6	10.4	10.1	10.9	10.9	11.3	12.0
FWHM (cm)	2.1	2.2	3.0	3.3	4.1	4.3	4.8	5.3
*10 MeV*								
$R_{100}\;(\text{mm})$	10.9	9.1	16.9	15.9	20.9	19.9	21.9	21.4
$R_{50}\;(\text{mm})$	30.9	31.4	35.7	36	37.4	37.5	37.0	38.0
$D_{x}\;(\%)$	1.6	1.2	1.6	1.1	1.5	1.1	1.4	1.1
Penumbra (mm)	5.8	6.3	8.3	8.6	10.6	10.9	10.9	12.1
FWHM (cm)	2.0	2.1	3.0	3.2	4.2	4.2	4.9	5.3
*15 MeV*								
$R_{100}\;(\text{mm})$	12.0	10.2	16.9	16.9	17.1	20.0	22.1	24.6
$R_{50}\;(\text{mm})$	41.3	41.4	49.9	49.9	55.4	55.4	58.4	58.4
$D_{x}\;(\%)$	3.9	2.9	3.9	2.6	3.9	2.6	3.7	2.6
Penumbra (mm)	4.7	4.9	5.8	6.1	7.2	7.2	8.8	8.9
FWHM (cm)	2.1	2.1	3.1	3.2	4.2	4.2	4.9	5.3

## IV. CONCLUSIONS

The dosimetric properties of small circular fields made with Cerrobend blocks and stainless steel commercial tubular applicators were compared in high‐energy radiotherapy electron beams. An overall similar behavior was observed by using the two different types of collimators in terms of PDDs and beam profiles. In particular, both systems are able to collimate the electron beam with comparable penumbra widths. Nevertheless, some differences have been found in output factors and bremsstrahlung background.

The OFs of the circular Cerrobend collimators have been found smaller than unity between 5 cm and 2 cm field size diameter, in agreement with the literature data referring to small size electron collimators. In the same conditions, the OFs of tubular applicators have been found higher than unity at 10 MeV and 15 MeV, thus allowing shorter treatment times at these energies. This advantage does not occur at low energy (6 MeV), where OFs for tubular applicators have been found lower than those for Cerrobend collimators in all the field sizes.

Tubular applicators are shown to produce a lower bremsstrahlung contribution in high‐energy irradiation conditions, leading to lower absorbed doses outside the clinical target volume. On the other hand, home‐made Cerrobend collimators are more flexible devices, allowing also the dose delivery by more complex specific beam shapes, other than the simply circular type.

## ACKNOWLEDGMENTS

The authors wish to thank “Fondazione Roma” for financial support and PTW‐Freiburg for providing the premarket microDiamond prototype and for helpful discussion.

## Supporting information

Supplementary Material
